# Associations of adipose and muscle tissue parameters at colorectal cancer diagnosis with long-term health-related quality of life

**DOI:** 10.1007/s11136-017-1539-z

**Published:** 2017-03-17

**Authors:** Eline H. van Roekel, Martijn J. L. Bours, Malou E. M. te Molder, José J. L. Breedveld-Peters, Steven W. M. Olde Damink, Leo J. Schouten, Silvia Sanduleanu, Geerard L. Beets, Matty P. Weijenberg

**Affiliations:** 1 Department of Epidemiology, GROW School for Oncology and Developmental Biology, Maastricht University, P.O. Box 616, 6200 MD Maastricht, The Netherlands; 2 Department of Surgery, NUTRIM School for Nutrition, Toxicology and Metabolism, Maastricht University Medical Center+, P.O. Box 5800, 6202 AZ Maastricht, The Netherlands; 3 Department of Internal Medicine, Division of Gastroenterology and Hepatology, GROW School for Oncology and Developmental Biology, Maastricht University Medical Center+, P.O. Box 5800, 6202 AZ Maastricht, The Netherlands; 4 Department of Surgery, GROW School for Oncology and Developmental Biology, Maastricht University Medical Center+, P.O. Box 5800, 6202 AZ Maastricht, The Netherlands

**Keywords:** Visceral adiposity, Muscle wasting, Sarcopenia, Health-related quality of life, Colorectal cancer

## Abstract

**Purpose:**

Increased visceral adiposity (visceral obesity) and muscle wasting (sarcopenia) at colorectal cancer (CRC) diagnosis, quantified by computed tomography (CT) image analysis, have been unfavorably associated with short-term clinical outcomes and survival, but associations with long-term health-related quality of life (HRQoL) have not been investigated. We studied associations of visceral adiposity, muscle fat infiltration, muscle mass, and sarcopenia at CRC diagnosis with HRQoL 2–10 years post-diagnosis.

**Methods:**

A cross-sectional study was conducted in 104 stage I‒III CRC survivors, diagnosed at Maastricht University Medical Center+, the Netherlands (2002–2010). Diagnostic CT images at the level of the third lumbar vertebra were analyzed to retrospectively determine visceral adipose tissue area (cm^2^); intermuscular adipose tissue area (cm^2^) and mean muscle attenuation (Hounsfield units) as measures of muscle fat infiltration; and skeletal muscle index (SMI, cm^2^/m^2^) as measure of muscle mass and for determining sarcopenia.

**Results:**

Participants showed a large variation in body composition parameters at CRC diagnosis with a mean visceral adipose tissue area of 136.1 cm^2^ (standard deviation: 93.4) and SMI of 47.8 cm^2^/m^2^ (7.2); 47% was classified as being viscerally obese, and 32% as sarcopenic. In multivariable linear regression models, associations of the body composition parameters with long-term global quality of life, physical, role and social functioning, disability, fatigue, and distress were not significant, and observed mean differences were below predefined minimal important differences.

**Conclusions:**

Although visceral obesity and sarcopenia are relatively common at CRC diagnosis, we found no significant associations of these parameters with long-term HRQoL in stage I–III CRC survivors.

**Electronic supplementary material:**

The online version of this article (doi:10.1007/s11136-017-1539-z) contains supplementary material, which is available to authorized users.

## Introduction

Globally, over 3.5 million individuals are living with a diagnosis of CRC in the past 5 years [1]. CRC patients often suffer from adverse effects of their disease and its treatment, leading to problems such as fatigue, bowel dysfunction, and distress [2–4]. These problems frequently persist for more than 5 years post-treatment and can severely impact the health-related quality of life (HRQoL) of CRC survivors in the long term [5]. Since the population of CRC survivors is steadily growing [6], it is a major research priority to identify characteristics of CRC patients with an increased risk of low long-term HRQoL [7], for this could enable development of targeted interventions to prevent HRQoL deterioration. In a recent systematic review, a comprehensive overview was provided on biopsychosocial factors associated with HRQoL of CRC survivors [8]. Strong evidence for an association with a lower HRQoL was found for several factors potentially associated with a more unfavorable body composition, including the presence of comorbidity, fatigue, and psychological distress, low levels of physical activity, low socioeconomic status, and shorter time since CRC diagnosis. The evidence was graded inconsistent for gender and cancer stage. In addition, a strong level of evidence was found for obesity being associated with worse HRQoL outcomes in CRC survivors. Indeed, previous prospective and cross-sectional studies have found that a higher body mass index (BMI) or the presence of obesity in comparison to other BMI categories in the period from 6 months until 10 years after CRC diagnosis was associated with a lower HRQoL in CRC survivors, in particular with a lower level of physical functioning [9–14]. Thus, specific characteristics of body composition of CRC patients at diagnosis (i.e., distribution and amount of adipose and muscle tissue) are likely to be associated with their health status, and therefore possibly also with long-term HRQoL in the post-diagnosis period. As increased adiposity is an established risk factor for CRC development [15], and muscle wasting can be an important consequence of cancer [16], a relatively high prevalence of these unhealthy body composition profiles can be expected in CRC patients at diagnosis.

Detailed and accurate body composition analysis of muscle tissue and different adipose tissue compartments at CRC diagnosis is possible through the use of images from routinely taken diagnostic computed tomography (CT) scans [17]. CT-based body composition analysis enables a highly accurate and precise quantification of muscle mass and different types of adipose tissue, which is to be preferred over more crude and inaccurate measures of body composition such as BMI [18]. As these CT scans are made as part of standard diagnostic procedures of cancer patients and therefore readily available for the majority of patients at the moment of diagnosis, this provides the opportunity to determine these parameters that are likely associated with long-term health outcomes. Previous research using CT-derived body composition parameters has observed associations of increased visceral adiposity (visceral obesity) and excessive muscle wasting (sarcopenia) at CRC diagnosis with worse short-term clinical outcomes and survival [19]. In particular, increased visceral adipose tissue has been associated with a higher rate of postoperative infections and/or complications [20–22], a longer postoperative hospital stay [20], and poorer disease-free survival [21, 23, 24]. In addition, parameters of skeletal muscle wasting (sarcopenia) have been associated with a longer length of hospital stay [25], more postoperative infections and/or complications [25, 26], increased chemotherapy toxicity [27], higher 30-day mortality [28], and worse recurrence-free [29] and overall survival [29–31]. Specifically, these studies found adverse effects of both reduced skeletal muscle mass [25, 27–31], and increased fat infiltration within muscle tissue (i.e., attenuation) [26, 31], which is an indicator of muscle quality and function [32, 33].

The above thus suggests that visceral adiposity and muscle wasting at CRC diagnosis can have negative effects on the frequency of postoperative complications and survival. However, whether these body composition parameters are also important for long-term HRQoL (≥2 years post-diagnosis) has not been investigated yet. As the occurrence of postoperative complications in CRC patients has been associated with worse global HRQoL [34], lower physical HRQoL, and more fatigue [35], 1–4 years post-diagnosis, it can be hypothesized that unfavorable muscle and adipose tissue parameters at CRC diagnosis are associated with long-term HRQoL. Further, sarcopenia at cancer diagnosis is an important factor associated with the development of cancer-related fatigue [36], and for this reason it can also be expected to negatively influence long-term quality of life in cancer patients [37]. In a small study in 50 stage IV CRC survivors, it has been found that sarcopenia at the moment of chemotherapy referral, determined by CT image analysis, was negatively associated with physical functioning, but not with other HRQoL outcomes [38]. However, this was a cross-sectional study with measures taken after diagnosis and in a select group of only stage IV CRC patients. In the light of future intervention programs aiming to prevent HRQoL deterioration in CRC survivors, it is of particular importance to study the associations of these body composition features at diagnosis in relation to long-term HRQoL. This would allow the development of interventions that can be provided at an early stage (e.g., nutritional support and exercise training before colorectal surgery to improve nutritional and functional status [39, 40]) and specifically targeted towards those CRC survivors in need of additional care. Thus, more research is necessary to determine whether parameters of visceral obesity and muscle wasting at CRC diagnosis are associated with long-term HRQoL outcomes in CRC survivors.

Therefore, our objective was to study associations of body composition parameters at CRC diagnosis with long-term HRQoL in CRC survivors, 2–10 years post-diagnosis. Specifically, we determined visceral adiposity, muscle fat infiltration, muscle mass, and sarcopenia through CT image analysis. We hypothesized that increased visceral adiposity and muscle fat infiltration, lower muscle mass, and sarcopenia at CRC diagnosis would be associated with lower long-term HRQoL.

## Methods

### Study design and participants

**Fig. 1:**
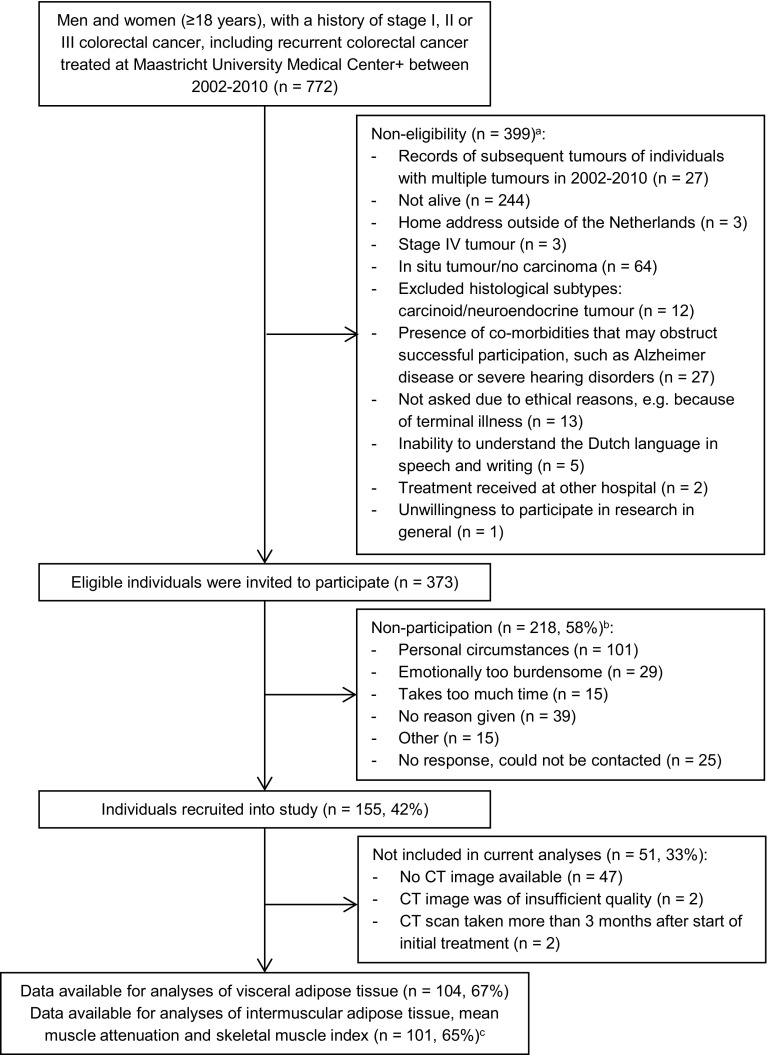
Flow diagram of inclusion of individuals into the cross-sectional part of the EnCoRe study and analyses presented in this paper. ^a^Reasons for non-eligibility are given in order of exclusion, totals do not add up because some exclusion criteria applied concurrently. ^b^Totals do not add up because some individuals reported multiple reasons for non-participation. ^c^Three computed tomography (CT) images excluded because skeletal muscle was not fully visible on CT image

Data from the cross-sectional part of the Energy for life after ColoRectal cancer (EnCoRe) study were used. Methods of the EnCoRe study have been described in detail elsewhere [41]. The cross-sectional part of the EnCoRe study was conducted in CRC survivors recruited 2–10 years post-diagnosis. Eligible individuals, i.e., persons diagnosed with and treated for stage I–III CRC between 2002 and 2010 at Maastricht University Medical Center+, the Netherlands, were preselected via the Netherlands Cancer Registry (managed by Comprehensive Cancer Centre the Netherlands). The main aim of the EnCoRe study is to investigate the associations of lifestyle factors with HRQoL in CRC survivors. Patients with stage IV CRC were therefore not included, since we hypothesized that their poor prognosis, and not lifestyle behavior, likely determines their HRQoL to the largest extent [41]. Participants were recruited between May 2012 and December 2013. Reasons for exclusion are shown in Fig. 1. The EnCoRe study had been approved by the Medical Ethics Committee of the Academic Hospital Maastricht and Maastricht University, the Netherlands. Written informed consent was obtained from all participants. No incentives were provided to participants.

### Data collection

When designing the EnCoRe study, a conceptual model was developed for studying HRQoL in CRC survivors [41], based on the International Classification of Functioning, Disability and Health (ICF) of the World Health Organization [42]. The ICF adopts a broad biopsychosocial definition of human functioning, including physical health components (body perspective) and the ability to perform daily activities and societal roles (individual and societal perspectives) [43]. Additionally, it enables the identification of environmental and personal factors and the presence of health conditions that can influence functioning. The developed conceptual model [41] was adapted for the current research questions to identify relevant variables to be included in data analyses (Online Resource 1, Supplementary Fig. 1).

#### Body composition variables

**Fig. 2:**
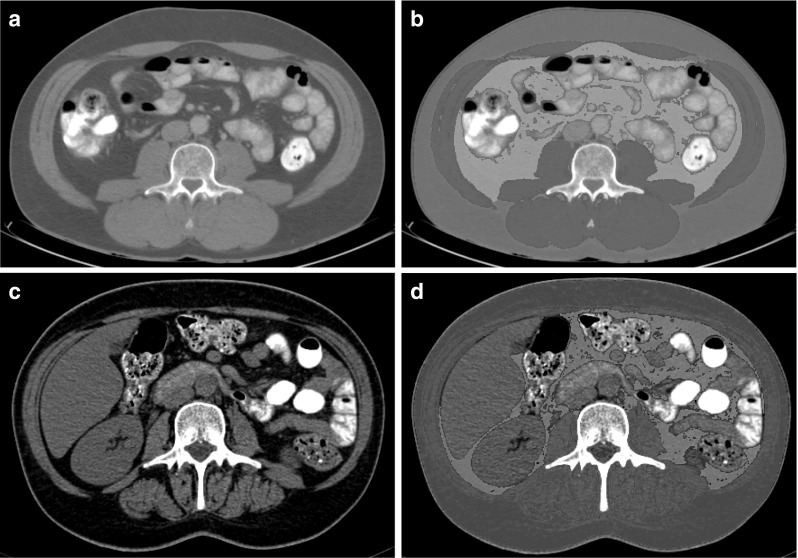
Computed tomography (CT) images showing the measurement of skeletal muscle and different types of adipose tissue at the level of the third lumbar vertebra (L3); **a** and **b** show the original and tagged (i.e., colored) CT images, respectively, of one included CRC survivor [man, 50 years old at CRC diagnosis, self-reported body mass index (BMI) at diagnosis: 27.6 kg/m^2^] with a relatively high skeletal muscle index (67.0 cm^2^/m^2^); area of visceral adipose tissue and intermuscular adipose tissue: 118.3 and 10.1 cm^2^, respectively; mean muscle attenuation: 35.9 Hounsfield units (HU); **c** and **d** show the original and tagged CT images, respectively, of one included CRC survivor (woman, 49 years old at CRC diagnosis, BMI at diagnosis: 21.0) with a relatively low skeletal muscle index (30.8 cm^2^/m^2^); area of visceral adipose tissue and intermuscular adipose tissue: 52.9 and 20.4 cm^2^, respectively; mean muscle attenuation: 27.2 HU. Within the tagged figure, *yellow* represents visceral adipose tissue; *blue* represents subcutaneous adipose tissue; *green* represents intermuscular adipose tissue; and *red* represents skeletal muscle. (Color figure online)

CT scans routinely taken at CRC diagnosis for diagnostic and staging purposes were obtained from medical records and used to quantify adipose and muscle tissue parameters (Fig. 2) [17, 31, 32]. CT scans taken more than 3 months after start of initial treatment were excluded (Fig. 1), as these were deemed to be unrepresentative of body composition at CRC diagnosis. The majority of included CT scans were taken before start of initial treatment (93%; range 0–185 days before start of treatment; 7% post-treatment; 4–36 days after start of treatment). According to published standard procedures [17, 31], the CT image at the level of the third lumbar vertebra (L3) was selected and analyzed with Slice-O-Matic software (version 5.0, TomoVision, Canada). It has been shown previously that cross-sectional areas of muscle and visceral fat tissue at a single cross-sectional image at the level of L3 correlate highly with total body muscle mass (Pearson’s *r* = 0.92) [44] and visceral fat volume (0.95 in men, 0.94 in women) [45], respectively. Relevant tissues were identified based on their anatomical features and the level of radiodensity in Hounsfield units (HU). The following HU ranges were applied: visceral adipose tissue, −150 to −50 HU; subcutaneous adipose tissue, −190 to −30 HU; intermuscular adipose tissue, −190 to −30 HU; and skeletal muscle, −29 to +150 HU [17]. The total cross-sectional area of visceral and intermuscular adipose tissue, and skeletal muscle was determined (cm^2^). The area of subcutaneous adipose tissue was not included in the analyses as this tissue was not fully visible (i.e., cutoff) in a substantial proportion of CT images (27%). CT image analyses were performed by two trained raters (MtM, MB). Intra-rater analyses showed excellent reproducibility for quantifying muscle and adipose tissues (absolute agreement type intraclass correlations: 1.00 for 9% duplicate analyses).

Based on visceral adipose tissue area, participants were classified as viscerally obese according to published gender-specific cutoff values for CT images at the level of L3–L4 (≥160 cm^2^ in men; ≥80 cm^2^ in women) [46]. In addition, the skeletal muscle index (SMI) was calculated by normalizing the total area of skeletal muscle for height squared (cm^2^/m^2^), as is conventional for this parameter [17, 31]. The SMI was used to determine the presence of sarcopenia, based on previously published threshold values for CT images at the level of L3 [31] and using self-reported weight at CRC diagnosis to retrospectively calculate BMI at diagnosis: <41 cm^2^/m^2^ in women (any BMI); <43 cm^2^/m^2^ in men with a BMI <25 kg/m^2^; and <53 cm^2^/m^2^ in men with a BMI ≥25 kg/m^2^. Further, mean muscle attenuation was determined as the mean radiodensity in HU of total skeletal muscle area. Next to intermuscular adipose tissue, the mean muscle attenuation is a measure of muscle fat infiltration and quality, with lower muscle attenuation indicating more muscle fat infiltration [31, 32].

At the time of HRQoL assessment, 2–10 years post-diagnosis, body height and weight were measured by trained personnel for calculation of BMI. In addition, as a measure of muscle function, isometric handgrip strength (kg) of the dominant hand was measured with a Jamar hand dynamometer (Sammons Preston Rolyan, USA), which has an excellent validity for measuring muscle strength in comparison with known weights (*r* > 0.99 [47]). The highest value of two measurements was used as a measure of maximum handgrip strength [48].

#### HRQoL outcomes

HRQoL outcomes were measured by self-report, 2–10 years post-diagnosis. Cancer-specific HRQoL was measured using the valid and reliable European Organization for the Research and Treatment of Cancer Quality of Life Questionnaire-Core 30 (EORTC QLQ-C30, version 3.0) [49, 50]. For the subscales global quality of life (2 items on a 7-point ordinal scale ranging from ‘Very poor’ to ‘Excellent’), and physical, role and social functioning (5, 2, and 2 items, respectively, all with a 5-point ordinal scale ranging from ‘Not at all’ to ‘Very much’), 100-point scores were calculated with higher scores indicating better HRQoL [51]. These subscales have been observed to have an acceptable internal consistency (Cronbach’s *α* > 0.70) [49, 52–55] and test–retest reliability (Pearson’s *r*: 0.58–0.75) [54]. Disability was assessed by the 12-item version of the ICF-based World Health Organization Disability Assessment Schedule II (WHODAS II; all items with a 5-point ordinal scale ranging from ‘None’ to ‘Extreme or cannot do’) [56], which has good test–retest reliability [intraclass correlation coefficient (ICC) = 0.98], internal consistency (Cronbach’s *α* = 0.98), and validity in different populations, including cancer survivors [57, 58]. Fatigue was assessed through the 20-item Checklist Individual Strength (CIS; all items with a 7-point ordinal scale ranging from ‘Yes, that is true’ to ‘No, that is not true’), which was originally developed and validated in chronic fatigue syndrome patients [59, 60], but has also been applied in cancer survivors [61]. The CIS has a good internal consistency (Cronbach’s *α* = 0.98) [59] and test–retest reliability (ICC = 0.81) [62]. The 14-item Hospital Anxiety and Depression Scale (HADS; all items with a 4-point ordinal scale with different answer options per item) was used to determine levels of distress (anxiety and/or depression) [63], which has adequate psychometric properties in cancer patients, including an adequate internal consistency type of reliability (Cronbach’s *α* > 0.80) [64]. Composite scores for disability (0–100) [57], fatigue (20–140), and distress (0–42) [64] were calculated, with higher scores indicating higher levels of disability, fatigue, and distress, respectively.

#### Other factors

Socio-demographic and clinical characteristics (gender, age at diagnosis, cancer stage, treatment, and tumor subsite) were collected through the Netherlands Cancer Registry. Education level was self-reported and the number of comorbidities, at HRQoL assessment, was assessed using the Self-Administered Comorbidity Questionnaire [65].

### Statistical analyses

Descriptive statistics for socio-demographic and clinical variables were calculated and compared between participants included in the analyses, participants without CT image data, and non-participants. Further, descriptive statistics of body composition and HRQoL variables were calculated by gender. Multivariable linear regression analyses were performed to determine mean differences (MDs) with 95% confidence intervals (CIs) in HRQoL outcomes between tertiles of CT-derived body composition variables using the first tertile as reference category. Total area of visceral and intermuscular adipose tissue and SMI were categorized into gender-specific tertiles. Tertiles of muscle attenuation were not gender-specific, as mean muscle attenuation did not differ between men and women (independent *t* test: *P* = 0.26). Testing for a linear trend was performed by including these variables as ordinal variables in the model. In addition, these parameters were included as continuous variables divided by their standard deviation (SD) in the study population, to calculate mean differences (MDs) in HRQoL scores per SD increase. Further, the dichotomous variables for the presence of visceral obesity and sarcopenia at CRC diagnosis were included to determine associations with HRQoL. Covariates included as potential confounding factors were selected a priori from our conceptual ICF model (Online Resource 1, Supplementary Fig. 1), comprising age at diagnosis, gender, number of comorbidities, cancer stage, chemotherapy treatment, and BMI at HRQoL assessment [the latter as proxy for self-reported BMI at diagnosis, because this variable was missing in 19% of participants and correlated highly with BMI at HRQoL assessment (*r* = 0.83)]. Based on regression diagnostics, the homoscedasticity assumption was not violated [66] and there was no multicollinearity as indicated by inspecting variance inflation factors [67]. Minimal important differences for all HRQoL outcomes were defined and based on published “medium” differences for the EORTC subscales [68], and 0.5 times the SD of the score for other outcomes [69] (disability, fatigue, and distress).

As additional analyses, we investigated associations of the CT-derived body composition parameters at CRC diagnosis with maximum handgrip strength at HRQoL assessment, as an objective measure of muscle strength and function [48]. Further, we performed stratified analyses by gender to compare results between men and women. To avoid over-interpretation of possible chance findings, results of these stratified analyses were reported only if a significant and meaningful (i.e., larger than the minimal important difference) association with multiple HRQoL outcomes between the highest or middle versus lowest tertile of the body composition parameter, or between the two groups of the dichotomous variables (visceral obesity, sarcopenia) was observed in men, but not in women, or vice versa. Analyses were performed using IBM SPSS Statistics (Version 22, IBM Corporation, United States of America), and *P* < 0.05 (two-tailed) was considered statistically significant.

## Results

### Participant characteristics

In total, 373 eligible CRC survivors were invited to participate and 155 were recruited (response rate: 42%; Fig. 1). A total of 51 participants (33%) were excluded from the current analyses for having no CT image at CRC diagnosis (*n* = 47), or a CT image of insufficient quality or taken more than 3 months after start of treatment (both *n* = 2). Three participants were further excluded from the analyses of muscle tissue parameters, due to skeletal muscle being not fully visible on the CT image. Non-participants had a higher mean age at diagnosis (67.8 years) than participating CRC survivors included in the analyses (64.3) and those without CT image data (64.6; Table 1). Further, compared to included survivors and non-participants, CRC survivors without CT image data had a higher time since diagnosis (differences: 1.5 and 1.0 years, respectively) and less often colon cancer and stage III CRC (differences >10%). In addition, included CRC survivors without CT image data were less often treated with chemotherapy, and reported less often a lower education level and more often ≥2 comorbidities than survivors included in the analyses (differences >10%).

**Table 1:** Socio-demographic and clinical characteristics of participants included in the current analyses, participants without computed tomography (CT) image data, and non-participants of the cross-sectional part of the EnCoRe study

Characteristic	Included in analyses (*n* = 104)	No available CT image data (*n* = 51)	Non-participants (*n* = 218)
Age at diagnosis (years), mean (SD)	64.3 (9.0)	64.6 (9.2)	67.8 (11.9)
Gender, n (%)			
Men	62 (59.6)	34 (66.7)	127 (58.3)
Women	42 (40.4)	17 (33.3)	91 (41.7)
Years since diagnosis, mean (SD)	5.2 (1.7)	6.7 (1.7)	5.7 (1.7)
Tumor subsite, n (%)			
Colon	60 (57.7)	23 (45.1)	132 (60.6)
Rectosigmoid	2 (1.9)	5 (9.8)	8 (3.7)
Rectum	42 (40.4)	23 (45.1)	78 (35.8)
Tumor stage^a^, n (%)			
I	27 (27.6)	16 (32.7)	55 (26.2)
II	33 (33.7)	21 (42.9)	81 (38.6)
III	38 (38.8)	12 (24.5)	74 (35.2)
Treatment with chemotherapy, n (%)	59 (56.7)	21 (41.2)	95 (43.6)
Treatment with radiotherapy, n (%)	38 (36.5)	22 (43.1)	66 (30.3)
Education level, n (%)			
Low	29 (27.9)	8 (17.0)	NA
Medium	31 (29.8)	21 (44.7)	
High	44 (42.3)	18 (38.3)	
Number of comorbid conditions, n (%)			
None	29 (27.9)	8 (16.0)	NA
1	30 (28.8)	8 (16.0)	
≥2	45 (45.3)	34 (68.0)	

*NA* not available, *SD* standard deviation

^a^Data missing for 16 survivors (6 participants included in analyses, 2 participants without CT image data, and 8 non-participants)

Included survivors (*n* = 104, 60% men, Table 1) were on average 64.3 years old (SD 9.0) at CRC diagnosis and their mean time since diagnosis was 5.2 years (SD 1.7) at the time of HRQoL assessment. The majority had a history of colon cancer (58%), whilst 40 and 2% had a rectum and rectosigmoid tumor, respectively. Nearly half (45%) reported having ≥2 comorbidities, and 49% were overweight (BMI 25–30 kg/m^2^) and 25% obese (BMI ≥30 kg/m^2^) at the time of HRQoL assessment. According to self-reported weight at CRC diagnosis, 43 and 12% could be classified as overweight and obese, respectively.

Data derived from CT images showed a large variation in body composition parameters at CRC diagnosis (Table 2), with a mean visceral adipose tissue area of 136.1 cm^2^ (SD 93.4), intermuscular adipose tissue area of 14.0 cm^2^ (8.8), muscle attenuation of 37.1 HU (8.7), and SMI of 47.8 cm^2^/m^2^ (7.2). Visceral adipose tissue area and SMI were on average higher in men, whilst intermuscular adipose tissue area was higher in women, and mean muscle attenuation was similar between genders. BMI at CRC diagnosis had a low to moderate correlation with the CT-derived parameters of body composition (Pearson’s *r*, ranging from −0.25 to 0.57). Based on published threshold values for visceral adipose tissue area [46], 49 survivors (47%) were classified as viscerally obese at CRC diagnosis (45% in men; 50% in women). In addition, using published SMI cutoff values [31], 29 participants (32%) were classified as sarcopenic (28% in men; 37% in women; data missing for 9 men due to unknown BMI at CRC diagnosis). In total, 12 participants (13%) were classified as being both viscerally obese and sarcopenic (16% in men; 10% in women).

**Table 2:** Descriptive statistics of body composition variables and health-related quality of life (HRQoL) outcomes of included colorectal cancer survivors by gender and tumor stage

	Gender				Tumor stage^a^						Total group (*n* = 104)	
	Men (*n* = 62)		Women (*n* = 42)		Stage I (*n* = 27)		Stage II (*n* = 33)		Stage III (*n* = 38)			
	Mean	(SD)	Mean	(SD)	Mean	(SD)	Mean	(SD)	Mean	(SD)	Mean	(SD)
Body composition variables^b^												
Visceral adipose tissue at diagnosis (cm^2^)	160.6	(100.6)	100.0	(67.7)	143.4	(90.4)	134.1	(91.4)	124.8	(92.7)	136.1	(93.4)
Intermuscular adipose tissue at diagnosis (cm^2^)	13.4	(9.2)	15.0	(8.1)	13.4	(7.3)	15.0	(8.5)	13.2	(9.2)	14.0	(8.8)
Mean muscle attenuation at diagnosis (HU)	37.8	(8.4)	36.2	(9.2)	36.2	(8.0)	36.0	(9.7)	38.8	(8.2)	37.1	(8.7)
Skeletal muscle index at diagnosis (cm^2^/m^2^)	51.3	(5.8)	42.6	(5.7)	47.6	(6.7)	47.4	(7.2)	48.2	(8.0)	47.8	(7.2)
Self-reported BMI at diagnosis (kg/m^2^)	25.6	(3.0)	25.8	(4.1)	26.0	(4.4)	25.5	(3.3)	25.6	(3.1)	25.7	(3.5)
Measured BMI at HRQoL assessment (kg/m^2^)	27.2	(3.3)	28.4	(5.5)	28.5	(5.2)	27.7	(4.6)	27.2	(3.5)	27.7	(4.3)
Maximum handgrip strength at HRQoL assessment (kg)	44.2	(9.1)	26.8	(5.7)	33.9	(10.3)	37.0	(12.5)	37.6	(11.0)	37.2	(11.6)
Health-related quality of life outcomes (scale)^c^												
Global quality of life (0–100)	79.0	(16.2)	77.2	(17.7)	74.1	(15.0)	79.8	(15.7)	80.7	(18.5)	78.3	(16.7)
Range (min–max)	25.0–100.0		41.7–100.0		50.0–100.0		33.3–100.0		25.0–100.0		25.0–100.0	
Physical functioning (0–100)	84.1	(19.1)	77.6	(21.0)	80.7	(18.6)	79.2	(19.9)	82.5	(21.8)	81.5	(20.0)
Range (min–max)	33.3–100.0		20.0–100.0		46.7–100.0		33.3–100.0		20.0–100.0		20.0–100.0	
Role functioning (0–100)	87.1	(23.7)	79.8	(27.2)	84.0	(21.9)	83.8	(25.2)	82.5	(29.2)	84.1	(25.3)
Range (min–max)	0.0–100.0		0.0–100.0		33.3–100.0		0.0–100.0		0.0–100.0		0.0–100.0	
Social functioning (0–100)	89.8	(18.4)	91.7	(13.4)	90.7	(13.3)	88.4	(19.3)	91.2	(17.2)	90.5	(16.5)
Range (min–max)	16.7–100.0		66.7–100.0		66.7–100.0		16.7–100.0		33.3–100.0		16.7–100.0	
Disability (0–100)	11.6	(14.7)	13.4	(16.6)	13.1	(11.9)	14.8	(18.2)	9.3	(15.5)	12.3	(15.4)
Range (min–max)	0.0–61.1		0.0–63.9		0.0–38.9		0.0–63.9		0.0–61.1		0.0–63.9	
Fatigue (20–140)	57.2	(26.6)	51.3	(26.7)	60.1	(23.8)	54.9	(29.7)	50.9	(26.7)	54.9	(26.6)
Range (min–max)	20.0–134.0		20.0–113.0		30.4–113.0		20.0–134.0		20.0–111.0		20.0–134.0	
Distress (0–21)	8.4	(6.1)	7.6	(6.2)	8.6	(4.5)	8.3	(7.0)	7.1	(6.4)	8.1	(6.1)
Range (min–max)	0.0–23.0		0.0–24.0		1.0–19.0		0.0–24.0		0.0–23.0		0.0–24.0	

*BMI* body mass index, *HU* Hounsfield units

^a^Data missing for 6 men

^b^Data missing for 3 participants (1 man with stage II, 1 man with tumor stage missing, and 1 woman with stage I) for skeletal muscle index, mean muscle attenuation, and intermuscular adipose tissue; and 20 participants (1 man with tumor stage missing, 2 men with stage I, 2 men with stage II, 5 men with stage III, 3 women with stage I, 6 women with stage II, and 1 woman with stage III) for self-reported BMI at diagnosis

^c^Higher scores indicate higher global quality of life, physical, role and social functioning, disability, fatigue, and distress. Data missing for 3 participants (1 man with stage II, 1 woman with stage I, and 1 woman with stage III) for disability, 2 women with stage II for fatigue, and 1 man with stage II for distress

Survivors with stage III CRC reported on average a better global quality of life, and less disability, fatigue, and distress than survivors with stage I/II CRC, but the differences were generally small. The other HRQoL outcomes scores were similar between tumor stages.

### Associations of adipose and muscle tissue parameters with HRQoL and handgrip strength

In multivariable linear regression models, no significant associations were observed of all CT-derived adipose and muscle tissue parameters with any of the self-reported HRQoL outcomes (all *P* values for testing the significance of all body composition variables included as independent variables in linear regression models ≥0.05; Table 3). Both models with body composition variables in tertiles and as continuous variables showed non-significant findings, indicating no linear or non-linear statistically significant associations were present. In addition, observed MDs were smaller than predefined minimal important differences for the HRQoL outcomes. Similarly, no significant associations were found of the body composition parameters with maximum handgrip strength (*P* ≥ 0.05; Table 4). Within stratified analyses, no differences in results between men and women were observed (Online resource 2, Supplementary Table 1).

**Table 3:** Mean differences in long-term health-related quality of life outcome scores in colorectal cancer survivors according to tertiles of area of visceral and intermuscular adipose tissue, mean muscle attenuation, and skeletal muscle index (SMI) at colorectal cancer diagnosis

	Global quality of life		Physical functioning		Role functioning		Social functioning		Disability		Fatigue		Distress	
	MD	95% CI	MD	95% CI	MD	95% CI	MD	95% CI	MD	95% CI	MD	95% CI	MD	95% CI
Visceral adipose tissue (cm^2^, *n* = 98)^a^														
T1	Ref		Ref		Ref		Ref		Ref		Ref		Ref	
T2	−7.6	−15.7, 0.6	−4.4	−14.8, 6.0	−2.0	−15.2, 11.2	−2.9	−11.3, 5.5	5.0	−3.0, 13.1	8.0	−5.8, 21.9	1.3	−1.8, 4.5
T3	−5.0	−15.3, 5.3	−4.2	−17.4, 9.0	1.3	−15.5, 18.1	−4.8	−15.5, 5.9	1.7	−8.5, 11.9	11.8	−5.8, 29.4	2.9	−1.1, 6.9
*P*_trend_	*0.33*		*0.52*		*0.88*		*0.37*		*0.73*		*0.18*		*0.16*	
Per SD increase	−1.2	−5.7, 3.3	2.6	−3.1, 8.2	4.0	−3.2, 11.2	0.8	−3.9, 5.4	−0.3	−4.8, 4.1	0.5	−7.2, 8.2	0.9	−0.8, 2.7
Visceral obesity^b^	2.3	−5.4, 10.1	4.0	−5.7, 13.7	8.6	−3.6, 20.8	−0.9	−8.8, 6.9	−2.3	−9.9, 5.2	0.7	−12.4, 13.7	−0.4	−3.4, 2.6
Intermuscular adipose tissue (cm^2^, *n* = 96)^a^														
T1	Ref		Ref		Ref		Ref		Ref		Ref		Ref	
T2	−2.3	−10.0, 5.4	−3.5	−13.2, 6.3	−4.9	−17.1, 7.3	2.0	−5.9, 9.9	5.2	−2.3, 12.7	0.6	−12.4, 13.7	0.8	−2.1, 3.8
T3	−2.6	−11.5, 6.3	−2.0	−13.2, 9.2	−2.8	−16.9, 11.3	3.3	−5.8, 12.4	3.3	−5.4, 12.0	−1.2	−16.5, 14.1	1.9	−1.5, 5.3
*P*_trend_	*0.54*		*0.69*		*0.66*		*0.46*		*0.40*		*0.89*		*0.27*	
Per SD increase	−0.9	−5.0, 3.1	−1.1	−6.2, 4.0	−1.3	−7.8, 5.1	2.1	−2.1, 6.2	0.1	−3.9, 4.2	0.1	−6.8, 7.1	1.1	−0.5, 2.6
Muscle attenuation (HU, *n* = 96)^a^														
T1	Ref		Ref		Ref		Ref		Ref		Ref		Ref	
T2	0.5	−8.1, 9.2	−4.3	−15.1, 6.6	−1.6	−15.4, 12.1	−0.6	−9.4, 8.2	0.4	−8.4, 9.2	3.7	−11.0, 18.3	−0.9	−4.3, 2.5
T3	−2.4	−11.6, 6.7	1.2	−10.3, 12.7	−2.9	−17.5, 11.6	−4.4	−13.7, 5.0	−1.6	−10.7, 7.6	2.7	−13.1, 18.5	−2.3	−5.9, 1.2
*P*_trend_	*0.57*		*0.76*		*0.69*		*0.33*		*0.71*		*0.76*		*0.18*	
Per SD increase	0.0	−3.9, 3.9	0.9	−4.0, 5.9	−1.1	−7.4, 5.1	−2.5	−6.5, 1.4	−0.8	−4.7, 3.0	−0.6	−7.6, 6.4	−1.1	−2.6, 0.4
SMI (cm^2^/m^2^, *n* = 96)^a^														
T1	Ref		Ref		Ref		Ref		Ref		Ref		Ref	
T2	0.6	−7.2, 8.5	3.5	−6.5, 13.4	6.1	−6.4, 18.7	−2.0	−10.1, 6.1	−3.2	−11.1, 4.6	2.2	−11.3, 15.7	−0.3	−3.3, 2.8
T3	3.7	−5.3, 12.7	4.8	−6.6, 16.1	4.9	−9.4, 19.2	1.2	−8.1, 10.4	−5.9	−15.0, 3.1	−3.5	−18.7, 11.7	−0.4	−3.9, 3.1
*P*_trend_	*0.42*		*0.40*		*0.48*		*0.82*		*0.19*		*0.65*		*0.82*	
Per SD increase	3.0	−1.5, 7.6	2.8	−2.9, 8.6	3.6	−3.7, 10.8	0.1	−4.6, 4.8	−4.6	−9.1, 0.0	−4.4	−12.1, 3.4	−0.7	−2.4, 1.1
Sarcopenia^c^	−3.3	−10.4, 3.8	−1.6	−10.4, 7.2	−1.1	−12.0, 9.7	2.3	−4.3, 8.8	2.3	−4.8, 9.4	−1.6	−13.4, 10.2	2.1	−0.6, 4.8

*P* values in italic indicate a significant level of *P* < 0.05

*CI* confidence interval, *HU* Hounsfield units, *MD* mean difference, *Ref* reference category, *SD* standard deviation

Adjusted for: gender, age at diagnosis (years), body mass index at health-related quality of life assessment (kg/m^2^), number of comorbidities (0/1/2+), cancer stage (I/II/III), and chemotherapy treatment (yes/no)

Scales are 0–100 (global quality of life, physical, role and social functioning, and disability), 20–140 (fatigue), and 0–21 (distress), with higher scores indicating higher global quality of life, physical, role and social functioning, disability, fatigue, and distress. A total of 3 participants had missing data for disability, 2 for fatigue, and 1 for distress. Predefined minimal important differences for these subscales are global quality of life, 10; physical functioning, 14; role functioning, 19; social functioning, 11; disability, 7.7; fatigue 13.3; and distress, 3.1

All associations of the body composition variables with the HRQoL outcomes that were tested within multivariable linear regression models and are shown in this table were not statistically significant (all *P* values ≥0.05)

^a^With gender-specific tertiles for visceral adipose tissue (men: T1, ≤100.3; T2, 102.7–196.5; T3, ≥199.2; women: T1, ≤58.7; T2, 59.2–131.0; T3, ≥139.1 cm^2^), intermuscular adipose tissue (men: T1, ≤8.3; T2, 8.8–14.2; T3, ≥14.3; women: T1, ≤9.6; T2, 9.7–17.8; T3, ≥17.9 cm^2^), and SMI (men: T1, ≤48.6; T2, 48.8–53.8; T3, ≥54.0; women: T1, ≤38.7; T2, 38.7–43.9; T3, ≥44.3 cm^2^/m^2^), and overall tertiles for muscle attenuation (T1, ≤33.6; T2, 33.6–41.6; T3, ≥42.0 Hounsfield units)

^b^Dichotomized based on published cutoff for visceral adipose tissue area [46]

^c^Dichotomized based on published cutoff for skeletal muscle index [31]; data missing for 9 participants due to missing data on body mass index at colorectal cancer diagnosis

**Table 4:** Mean differences in long-term maximum handgrip strength in colorectal cancer survivors according to tertiles of area of visceral and intermuscular adipose tissue, mean muscle attenuation, and skeletal muscle index (SMI) at colorectal cancer diagnosis

	Maximum handgrip strength	
	MD	95% CI
Visceral adipose tissue (cm^2^, *n* = 98)^a^		
T1	Ref	
T2	1.9	−1.7, 5.4
T3	2.9	−1.6, 7.4
*P*_trend_	*0.20*	
Per SD increase	1.5	−0.5, 3.4
Visceral obesity^b^	3.9	0.7, 7.2
Intermuscular adipose tissue (cm^2^, *n* = 96)^a^		
T1	Ref	
T2	0.4	−3.0, 3.7
T3	−1.7	−5.5, 2.2
*P*_trend_	*0.42*	
Per SD increase	−0.6	−2.4, 1.2
Muscle attenuation (HU, *n* = 96)^a^		
T1	Ref	
T2	1.7	−2.0, 5.4
T3	2.0	−1.9, 6.0
*P*_trend_	*0.33*	
Per SD increase	1.2	−0.5, 2.9
SMI (cm^2^/m^2^, *n* = 96)^a^		
T1	Ref	
T2	1.3	−2.1, 4.7
T3	−0.5	−4.4, 3.4
*P*_trend_	*0.83*	
Per SD increase	1.0	−1.0, 3.0
Sarcopenia^c^	−0.7	−3.7, 2.3

*P* values in italic indicate a significant level of *P* < 0.05

*CI* confidence interval, *HU* Hounsfield units, *MD* mean difference, *Ref* reference category, *SD* standard deviation, *SMI* skeletal muscle index

Adjusted for: gender, age at diagnosis (years), body mass index at health-related quality of life assessment (kg/m^2^), number of comorbidities (0/1/2+), cancer stage (I/II/III), and chemotherapy treatment (yes/no)

^a^With gender-specific tertiles for visceral adipose tissue (men: T1, ≤100.3; T2, 102.7–196.5; T3, ≥199.2; women: T1, ≤58.7; T2, 59.2–131.0; T3, ≥139.1 cm^2^), intermuscular adipose tissue (men: T1, ≤8.3; T2, 8.8–14.2; T3, ≥14.3; women: T1, ≤9.6; T2, 9.7–17.8; T3, ≥17.9 cm^2^), and SMI (men: T1, ≤48.6; T2, 48.8–53.8; T3, ≥54.0; women: T1, ≤38.7; T2, 38.7–43.9; T3, ≥44.3 cm^2^/m^2^), and overall tertiles for muscle attenuation (T1, ≤33.6; T2, 33.6–41.6; T3, ≥42.0 Hounsfield units)

^b^Dichotomized based on published cutoff for visceral adipose tissue area [46]

^c^Dichotomized based on cutoff [31]; data missing for 9 participants due to missing data on body mass index at colorectal cancer

## Discussion

To our knowledge, this is the first study investigating the associations of CT-derived muscle and adipose tissue parameters with long-term HRQoL in stage I–III CRC survivors, 2–10 years post-diagnosis. In contrast to our prior hypotheses, we observed no statistically significant associations of visceral adiposity, muscle fat infiltration, and muscle mass at CRC diagnosis with investigated long-term HRQoL outcomes. In addition, even though we observed that a substantial proportion of CRC survivors were classified as viscerally obese and sarcopenic at CRC diagnosis, we did not observe significant associations of these unfavorable body composition profiles with long-term HRQoL. All observed mean differences were below predefined minimal important differences, indicating that the differences observed are small and not likely to be clinically relevant.

Our results suggest that an unhealthy body composition at CRC diagnosis might not be an important determinant of long-term HRQoL in CRC survivors. Although increased visceral adiposity and muscle wasting may negatively affect clinical outcomes and functioning of CRC patients shortly after diagnosis and treatment [19], long-term CRC survivors may recover in terms of functioning and HRQoL. Prospective studies are needed to study associations of body composition at CRC diagnosis with short- and long-term HRQoL and test this hypothesis. Statistical power may have been limited for detecting real associations due to our relatively small sample size, but all observed mean differences in HRQoL outcomes were smaller than predefined minimal important differences. This indicates that observed associations of body composition parameters with HRQoL were not clinically relevant in our sample of long-term CRC survivors. Furthermore, recovery of functioning in the longer term was confirmed by our data, as we observed no significant associations of these body composition parameters at diagnosis with maximum handgrip strength (a measure of muscle strength and function) 2–10 years post-diagnosis. Thus, instead of their body composition at CRC diagnosis, other characteristics, such as comorbidities and level of physical activity during CRC survivorship, could be more relevant determinants of HRQoL in CRC survivors [8, 70]. It is important to note, however, that our study population did not include stage IV CRC survivors who have a worse prognosis. A previous study in a small sample of 50 stage IV CRC patients observed significant cross-sectional associations of CT-derived sarcopenia at referral for potential chemotherapy treatment (time since diagnosis not reported) with physical functioning, but not with other HRQoL outcomes [38]. We did not observe a significant association of sarcopenia at CRC diagnosis with physical functioning or any other HRQoL outcome. This difference is likely due to differences between this previous study and our study, as stage IV CRC patients were not included and measurements were conducted at different time points in our study (CT scans at diagnosis and HRQoL ≥2 years post-diagnosis).

Comparison of our results with findings from previous studies in CRC patients is complicated by heterogeneity in methods of CT image analyses used (e.g., analysis level and/or software), and by differences between study populations (e.g., stage and ethnicity). A previous study in US [71] stage I–IV rectal cancer patients reported a mean visceral adipose tissue area of 108.0 cm^2^, assessed through analysis of CT images at the level of the umbilicus (L3–L4). This is lower than the mean value we observed (136.1 cm^2^), which is likely due to differences between study populations. Previous studies in CRC patients have observed a large range in prevalence of visceral obesity of 17.5–61.4%, using a variety of CT-based analysis protocols and cutoffs [19]. We applied recently published threshold values for CT-based analysis of visceral adipose tissue area at the level of L3–L4 in gastrointestinal cancer (including CRC) patients [46], and observed a prevalence of 47% in our study population. For SMI and muscle attenuation, our results (mean SMI 51.3 cm^2^/m^2^ in men, 42.6 cm^2^/m^2^ in women; muscle attenuation: 37.8 HU in men, 36.2 HU in women) are comparable with averages reported in previous studies in stage I–IV CRC patients with analysis of CT images at L3 (range in mean SMI: 51.5–57.5 cm^2^/m^2^ in men, and 40.7–46.5 cm^2^/m^2^ in women [25, 27, 28, 31]; and mean muscle attenuation of 35.5 HU in men, and 36.2 HU in women [31]). Similarly, the proportion of sarcopenic patients at CRC diagnosis we observed in our sample (32%) falls within the range reported in previous studies using CT-based analysis at the level of L3 (25–48%) [25, 28, 29, 31].

An important strength of our study was the use of CT image analyses, which enabled us to accurately and precisely determine adipose and muscle tissue at CRC diagnosis. As parameters of muscle wasting, we determined SMI as a measure for muscle mass, and mean muscle attenuation and intermuscular adipose tissue area as measures for muscle fat infiltration and quality. Low correlations of the latter parameters with SMI in our study population indicate that these measure distinct features of the muscle wasting process (both *r* = 0.0). CT-based analysis is regarded superior to anthropometric measures of body composition including BMI, which cannot differentiate between different types of body tissues [18]. Correlations between BMI at CRC diagnosis with CT-derived parameters of body composition were low to moderate in our study, illustrating the differences between BMI and these measures. By retrospective analysis of CT images from CRC survivors in our cross-sectional sample, we were able to study longitudinal associations of body composition at CRC diagnosis with HRQoL, 2–10 years later. However, a limitation of our cross-sectional design is the possibility of selection bias. Based on the differences observed between participants and non-participants, it is likely that survivors with a more unfavorable body composition at CRC diagnosis and lower long-term HRQoL were less likely to participate, which could have attenuated our associations. Another limitation was that no CT images were available for a substantial proportion of CRC survivors, probably due to changes in diagnostic procedures over the years, which was supported by the observation that fewer CT images were available from CRC survivors diagnosed longer ago. We additionally observed that participants with available CT scans had less often ≥2 comorbidities that those without a CT scan, which also suggests that a more ‘healthy’ subset of CRC survivors was included in the current study. This could have further attenuated our findings. In addition, included CRC survivors had a wide range in time since diagnosis (2–10 years), but we could not analyze the associations between body composition variables and HRQoL stratified for time since diagnosis due to our limited sample size. The occurrence of a response shift (i.e., change in internal standards, personal values, and the conceptualization of HRQoL due to the confrontation with a life-threatening disease such as cancer [72]) in the post-diagnosis period cannot be excluded and may have influenced our findings. Future prospective studies are needed in which CT scans are collected in a standardized way at CRC diagnosis in a consecutive sample of CRC patients, and survivors are followed up with repeated measurements of HRQoL. This will enable an investigation of the associations of body composition parameters at CRC diagnosis with the development of HRQoL during the CRC survivorship trajectory and will provide insight into the temporality of associations. Furthermore, we were unable to determine the presence of sarcopenic obesity at CRC diagnosis [30, 73], because data to determine BMI at CRC diagnosis were not available in a substantial proportion of participants (19%).

In conclusion, although parameters of increased visceral adiposity and excessive muscle wasting at CRC diagnosis have been associated with worse short-term clinical outcomes and survival, our findings suggest that these parameters may not be associated with long-term HRQoL in our sample of stage I–III CRC survivors. This suggests that interventions targeting CRC patients with an unhealthy body composition at diagnosis could be favorable towards improving short-term clinical outcomes and survival, but might not be relevant for improving long-term HRQoL. Prospective studies are needed to further investigate longitudinal associations of body composition at CRC diagnosis with HRQoL in CRC survivors. Since we found that muscle wasting and visceral adiposity were relatively common in our population of included stage I–III CRC patients, we think it is important that interventions are being developed and tested to reduce visceral adiposity and inhibit muscle wasting in CRC patients. Studying the effects of these interventions on short- and long-term HRQoL and other relevant outcomes, preferably in randomized controlled trials, will provide more insight into the potential causality of associations of muscle wasting and visceral adiposity with short- and long-term health and well-being of CRC survivors.

## Electronic supplementary material

Below is the link to the electronic supplementary material.

Supplementary material 1 (PDF 75 KB)

Supplementary material 2 (PDF 286 KB)
