# Supplementary material for: Associations of adipose and muscle tissue parameters at colorectal cancer diagnosis with long-term health-related quality of life
Source: Qual Life Res. 2017 Mar 17;26(7):1745–59. doi: 10.1007/s11136-017-1539-z (PMC5486890; doi:10.1007/s11136-017-1539-z)
Supplement: Supplementary file 1 — Supplementary material 1 (PDF 75 KB) [file 11136_2017_1539_MOESM1_ESM.pdf]

Associations of adipose and muscle tissue parameters at colorectal cancer diagnosis with long-term health-related quality of life

Eline H. van Roekel[1], Martijn J.L. Bours[1], Malou E.M. te Molder[1], José J.L. Breedveld-Peters[1], Steven W.M. Olde Damink[2], Leo J. Schouten[1], Silvia Sanduleanu[3], Geerard L Beets[4] and Matty P. Weijenberg[1]

Corresponding author: Eline H. van Roekel, Maastricht University, Department of Epidemiology, GROW School for Oncology and Developmental Biology, P.O. Box 616, 6200 MD Maastricht, The Netherlands. Phone: +31 43 38 83428. Fax: +31 43 38 84128. E-mail: eline.vanroekel@maastrichtuniversity.nl.

Quality of Life Research

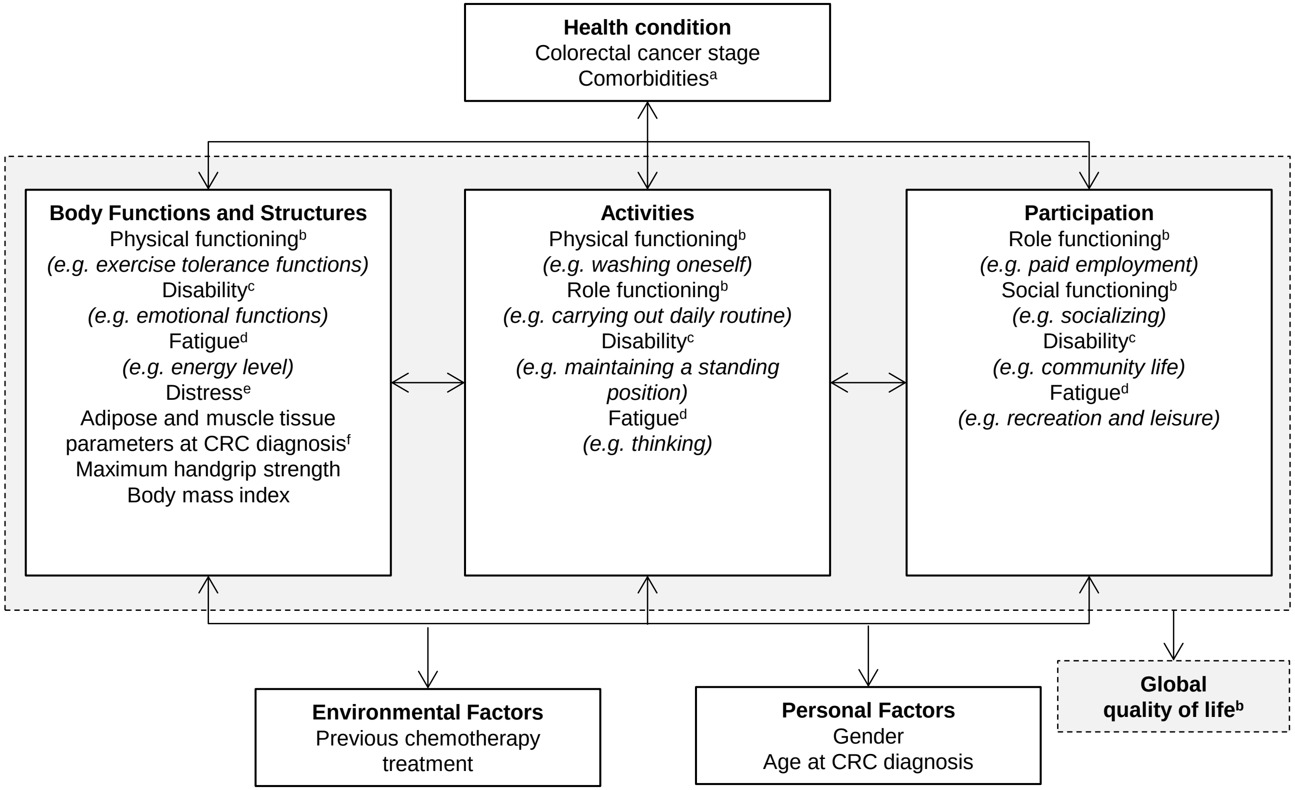

Supplementary Figure 1: Conceptual model, based on the International Classification of Functioning, Disability and Health (ICF), showing relevant factors for studying association of adipose and muscle tissue parameters at colorectal cancer (CRC) diagnosis with long-term health-related quality of life (HRQoL) outcomes in CRC survivors, which were measured within the cross-sectional part of the Energy for life after ColoRectal cancer study and included in the current analyses.

Footnotes:

The ICF is a biopsychosocial framework of health and functioning that comprehensively describes and classifies functioning of an individual or a group of individuals in a certain health state by differentiating between body functions and structures, activities, and participation, in the context of environmental and personal factors (barriers/facilitators). Certain HRQoL outcomes are shown within multiple ICF domains for functioning (e.g., Physical functioning is shown in both ‘Body functions and Structures’ and ‘Activities’), because meaningful concepts contained in these measures were linked to specific ICF categories within these different domains of functioning (e.g. ‘exercise tolerance functions’ and ‘washing oneself’).

Measurement instruments/techniques: [a]Self-administered Comorbidity Questionnaire; [b]European Organization for the Research and Treatment of Cancer Quality of Life Questionnaire-Core 30 (EORTC QLQ-C30); [c]12-item World Health Organization Disability Assessment Schedule II; [d]Checklist Individual Strength; [e]Hospital Anxiety and Depression Scale; [f]Determined from analyses of CT images at the level of the third lumbar vertebra (L3).
