# Supplementary material for: Associations of adipose and muscle tissue parameters at colorectal cancer diagnosis with long-term health-related quality of life
Source: Qual Life Res. 2017 Mar 17;26(7):1745–59. doi: 10.1007/s11136-017-1539-z (PMC5486890; doi:10.1007/s11136-017-1539-z)
Supplement: Supplementary file 2 — Supplementary material 2 (PDF 286 KB) [file 11136_2017_1539_MOESM2_ESM.pdf]

Associations of adipose and muscle tissue parameters at colorectal cancer diagnosis with long-term health-related quality of life

Eline H. van Roekel[1], Martijn J.L. Bours[1], Malou E.M. te Molder[1], José J.L. Breedveld-Peters[1], Steven W.M. Olde Damink[2], Leo J. Schouten[1], Silvia Sanduleanu[3], Geerard L Beets[4] and Matty P. Weijenberg[1]

Corresponding author: Eline H. van Roekel, Maastricht University, Department of Epidemiology, GROW School for Oncology and Developmental Biology, P.O. Box 616, 6200 MD Maastricht, The Netherlands. Phone: +31 43 38 83428. Fax: +31 43 38 84128. E-mail: eline.vanroekel@maastrichtuniversity.nl.

Quality of Life Research

Supplementary Table 1. Mean differences[a] in long-term health-related quality of life outcome scores[b] in colorectal cancer survivors according to tertiles of area of visceral and intermuscular adipose tissue, mean muscle attenuation, and skeletal muscle index (SMI) at colorectal cancer diagnosis, stratified by gender

	**Global quality of life**		**Physical functioning**		**Role functioning**		**Social functioning**		**Disability**		**Fatigue**		**Distress**	
	MD	95% CI	MD	95% CI	MD	95% CI	MD	95% CI	MD	95% CI	MD	95% CI	MD	95% CI
Visceral adipose tissue (cm^2)[c]														
Total group (n=98)														
T1	ref		ref		ref		ref		ref		ref		ref	
T2	-7.6	-15.7, 0.6	-4.4	-14.8, 6.0	-2.0	-15.2,11.2	-2.9	-11.3, 5.5	5.0	-3.0, 13.1	8.0	-5.8, 21.9	1.3	-1.8, 4.5
T3	-5.0	-15.3, 5.3	-4.2	-17.4, 9.0	1.3	-15.5,18.1	-4.8	-15.5, 5.9	1.7	-8.5, 11.9	11.8	-5.8, 29.4	2.9	-1.1, 6.9
P_{trend}	*0.33*		*0.52*		*0.88*		*0.37*		*0.73*		*0.18*		*0.16*	
Per SD increase	-1.2	-5.7, 3.3	2.6	-3.1, 8.2	4.0	-3.2, 11.2	0.8	-3.9, 5.4	-0.3	-4.8, 4.1	0.5	-7.2, 8.2	0.9	-0.8, 2.7
Visceral obesity[d]	2.3	-5.4, 10.1	4.0	-5.7, 13.7	8.6	-3.6, 20.8	-0.9	-8.8, 6.9	-2.3	-9.9, 5.2	0.7	-12.4, 13.7	-0.4	-3.4, 2.6
Men (n=56)														
T1	ref		ref		ref		ref		ref		ref		ref	
T2	-3.8	-14.0, 6.3	-0.3	-13.6, 13.0	4.4	-11.6, 20.4	-3.7	-16.0, 8.6	1.8	-8.1, 11.8	-2.2	-20.8, 16.4	-0.1	-4.5, 4.3
T3	-3.8	-16.2, 8.5	-3.7	-19.9, 12.6	3.8	-15.7, 23.4	-6.2	-21.2, 8.8	3.7	-8.4, 15.7	8.2	-14.5, 30.8	2.4	-3.0, 7.7
P_{trend}	*0.52*		*0.65*		*0.68*		*0.40*		*0.54*		*0.49*		*0.39*	
Per SD increase	-0.8	-6.0, 4.4	0.8	-5.9, 7.6	4.0	-4.1, 12.1	-0.2	-6.5, 6.1	1.6	-3.5, 6.8	0.4	-9.2, 9.9	0.4	-1.9, 2.7
Visceral obesity[d]	-0.4	-9.3, 8.5	1.7	-9.9, 13.3	3.9	-10.0, 17.9	-3.8	-14.5, 6.9	-0.8	-9.4, 7.9	5.5	-10.8, 21.7	-0.2	-4.0, 3.7
Women (n=42)														
T1	ref		ref		ref		ref		ref		ref		ref	
T2	-9.8	-25.2, 5.6	-11.3	-30.2, 7.6	-15.4	-40.8, 10.1	-1.5	-13.3, 10.3	12.1	-4.0, 28.2	29.1	6.2, 52.1	4.2	-1.0, 9.4
T3	2.0	-21.2, 25.2	-7.5	-36.0, 21.0	-9.1	-47.4, 29.2	-0.1	-17.9, 17.7	2.4	-21.8, 26.6	31.4	-3.2, 66.1	4.8	-3.0, 12.6
P_{trend}	*0.94*		*0.56*		*0.59*		*0.98*		*0.80*		*0.07*		*0.20*	
Per SD increase	1.2	-12.5, 15.0	-1.1	-17.4, 15.2	-0.0	-22.0, 22.0	3.8	-6.1, 13.6	-1.9	-16.0, 12.1	11.8	-9.4, 32.9	2.9	-1.5, 7.3
Visceral obesity[d]	13.2	-3.2, 29.5	7.6	-12.4, 27.6	15.9	-10.7, 42.5	8.2	-3.7, 20.1	-6.3	-23.5, 10.9	-2.9	-29.4, 23.7	0.1	-5.5, 5.7
Intermuscular adipose tissue (cm^2)[c]														
Total group (n=96)														
T1	ref		ref		ref		ref		ref		ref		ref	
T2	-2.3	-10.0, 5.4	-3.5	-13.2, 6.3	-4.9	-17.1, 7.3	2.0	-5.9, 9.9	5.2	-2.3, 12.7	0.6	-12.4,13.7	0.8	-2.1, 3.8
T3	-2.6	-11.5, 6.3	-2.0	-13.2, 9.2	-2.8	-16.9,11.3	3.3	-5.8, 12.4	3.3	-5.4, 12.0	-1.2	-16.5,14.1	1.9	-1.5, 5.3
P_{trend}	*0.54*		*0.69*		*0.66*		*0.46*		*0.40*		*0.89*		*0.27*	
Per SD increase	-0.9	-5.0, 3.1	-1.1	-6.2, 4.0	-1.3	-7.8, 5.1	2.1	-2.1, 6.2	0.1	-3.9, 4.2	0.1	-6.8, 7.1	1.1	-0.5, 2.6
Men (n=55)														
T1	ref		ref		ref		ref		ref		ref		ref	
T2	0.5	-10.1, 11.1	-8.9	-22.3, 4.5	-2.1	-18.5, 14.2	7.4	-5.2, 20.0	10.0	0.2, 19.8	2.1	-17.6, 21.7	0.9	-3.8, 5.5
T3	1.6	-11.3, 14.5	0.2	-16.1, 16.5	9.8	-10.1, 29.6	9.4	-5.9, 24.8	3.6	-8.3, 15.5	-0.0	-23.8, 23.8	1.1	-4.5, 6.7
P_{trend}	*0.80*		*0.99*		*0.33*		*0.22*		*0.56*		*1.00*		*0.70*	
Per SD increase	2.6	-3.1, 8.2	1.9	-5.5, 9.3	5.9	-2.9, 14.7	3.7	-3.1, 10.6	-0.6	-6.1, 4.9	-1.5	-12.0, 9.1	0.6	-1.8, 3.1
Women (n=41)														
T1	ref		ref		ref		ref		ref		ref		ref	
T2	0.3	-13.7, 14.2	7.3	-9.1, 23.7	-0.2	-21.7, 21.2	2.1	-7.9, 12.1	-3.8	-18.0, 10.4	-1.6	-23.2, 20.1	0.5	-4.1, 5.1
T3	-3.5	-18.2, 11.2	-3.6	-20.8, 13.7	-12.4	-35.1, 10.2	2.5	-8.0, 13.1	4.1	-10.9, 19.1	-4.0	-27.8, 19.8	1.9	-3.0, 6.7
P_{trend}	*0.65*		*0.79*		*0.30*		*0.60*		*0.66*		*0.73*		*0.44*	
Per SD increase	-3.2	-10.4, 4.0	-3.8	-12.4, 4.8	-7.9	-19.0, 3.2	2.8	-2.3, 7.9	1.7	-5.8, 9.3	1.0	-10.7, 12.6	1.2	-1.2, 3.6

Muscle attenuation (HU)[c]														
Total group (n=96)														
T1	ref		ref		ref		ref		ref		ref		ref	
T2	0.5	-8.1, 9.2	-4.3	-15.1, 6.6	-1.6	-15.4,12.1	-0.6	-9.4, 8.2	0.4	-8.4, 9.2	3.7	-11.0,18.3	-0.9	-4.3, 2.5
T3	-2.4	-11.6, 6.7	1.2	-10.3, 12.7	-2.9	-17.5,11.6	-4.4	-13.7, 5.0	-1.6	-10.7, 9.2	2.7	-13.1,18.5	-2.3	-5.9, 1.2
P_{trend}	*0.57*		*0.76*		*0.69*		*0.33*		*0.71*		*0.76*		*0.18*	
Per SD increase	0.0	-3.9, 3.9	0.9	-4.0, 5.9	-1.1	-7.4, 5.1	-2.5	-6.5, 1.4	-0.8	-4.7, 3.0	-0.6	-7.6, 6.4	-1.1	-2.6, 0.4
Men (n=55)														
T1	ref		ref		ref		ref		ref		ref		Ref	
T2	6.3	-5.3, 18.0	2.8	-12.6, 18.1	5.1	-13.4, 23.6	-2.7	-17.0, 11.6	-12.7	-23.8, -1.5	-2.5	-24.3, 19.4	-4.1	-9.3, 1.0
T3	0.7	-11.2, 12.5	4.8	-10.9, 20.4	-2.7	-21.5, 16.1	-5.8	-20.3, 8.8	-12.4	-23.6, -1.2	2.6	-19.7, 24.8	-4.7	-9.8, 0.5
P_{trend}	*0.96*		*0.54*		*0.69*		*0.42*		*0.05*		*0.77*		*0.10*	
Per SD increase	-0.9	-6.7, 4.8	1.9	-5.5, 9.4	-3.7	-12.6, 5.3	-3.4	-10.2, 3.5	-5.0	-10.5, 0.5	0.7	-9.9, 11.3	-2.4	-4.8, 0.1
Women (n=41)														
T1	ref		ref		ref		ref		ref		ref		ref	
T2	-5.8	-20.4, 8.7	-15.6	-32.3, 1.1	-7.3	-30.3, 15.7	1.6	-8.8, 12.0	13.2	-1.4, 27.8	10.9	-12.3, 34.0	1.6	-3.2, 6.4
T3	-7.3	-23.4, 8.7	-6.7	-25.1, 11.7	-3.9	-29.3, 21.5	-3.2	-14.6, 8.3	9.2	-6.8, 25.2	6.0	-20.5, 32.5	0.3	-5.0, 5.7
P_{trend}	*0.36*		*0.56*		*0.78*		*0.54*		*0.30*		*0.70*		*0.94*	
Per SD increase	-0.3	-6.6, 6.0	-0.9	-8.4, 6.6	-0.0	-9.9, 9.8	-3.0	-7.4, 1.3	2.2	-4.3, 8.6	-1.4	-12.6, 9.7	0.1	-2.0, 2.2
SMI (cm^2/m^2)[c]														
Total group (n=96)														
T1	ref		ref		ref		ref		ref		ref		ref	
T2	0.6	-7.2, 8.5	3.5	-6.5, 13.4	6.1	-6.4, 18.7	-2.0	-10.1, 6.1	-3.2	-11.1, 4.6	2.2	-11.3,15.7	-0.3	-3.3, 2.8
T3	3.7	-5.3, 12.7	4.8	-6.6, 16.1	4.9	-9.4, 19.2	1.2	-8.1, 10.4	-5.9	-15.0, 3.1	-3.5	-18.7,11.7	-0.4	-3.9, 3.1
P_{trend}	*0.42*		*0.40*		*0.48*		*0.82*		*0.19*		*0.65*		*0.82*	
Per SD increase	3.0	-1.5, 7.6	2.8	-2.9, 8.6	3.6	-3.7, 10.8	0.1	-4.6, 4.8	-4.6	-9.1, 0.0	-4.4	-12.1, 3.4	-0.7	-2.4, 1.1
Sarcopenia[e]	-3.3	-10.4, 3.8	-1.6	-10.4, 7.1	-1.1	-12.0, 9.7	2.3	-4.3, 8.8	2.3	-4.8, 9.4	-1.6	-13.4, 10.2	2.1	-0.6, 4.8
Men (n=55)														
T1	ref		ref		ref		ref		ref		ref		ref	
T2	2.0	-8.3, 12.2	2.4	-11.0, 15.7	4.6	-11.3, 20.6	0.2	-12.2, 12.6	-3.3	-13.2, 6.5	4.1	-14.8, 23.0	3.0	-1.4, 7.3
T3	-0.9	-12.4, 10.5	-1.0	-15.9, 13.9	-3.4	-21.3, 14.4	-2.2	-16.1, 11.7	0.2	-10.8, 11.2	2.7	-18.5, 23.9	3.2	-1.6, 8.1
P_{trend}	*0.89*		*0.90*		*0.73*		*0.76*		*1.00*		*0.79*		*0.17*	
Per SD increase	2.6	-3.0, 8.2	1.3	-6.1, 8.7	-0.4	-9.3, 8.5	-0.8	-7.7, 6.1	-2.6	-8.1, 2.8	-3.2	-13.6, 7.3	0.4	-2.0, 2.9
Sarcopenia[e]	-5.8	-15.2, 3.6	-4.0	-16.2, 8.2	1.2	-12.7, 15.0	0.9	-9.4, 11.2	1.5	-7.8, 10.7	3.9	-13.3, 21.1	1.0	-3.1, 5.2
Women (n=41)														
T1	ref		ref		ref		ref		ref		ref		ref	
T2	-2.3	-16.6, 12.0	2.9	-14.2, 20.0	4.3	-18.4, 27.1	-7.1	-17.1, 2.9	-3.5	-19.0, 12.0	5.7	-16.3, 27.7	-3.0	-7.6, 1.6
T3	8.0	-8.8, 24.8	12.4	-7.7, 32.5	11.3	-15.4, 38.0	0.8	-11.0, 12.6	-14.4	-33.5, 4.7	-13.7	-38.7, 11.3	-5.1	-10.5, 0.2
P_{trend}	*0.41*		*0.23*		*0.39*		*0.92*		*0.14*		*0.34*		*0.05*	
Per SD increase	3.6	-5.2, 12.4	5.6	-4.9, 16.1	8.3	-5.4, 21.9	-0.4	-6.8, 5.9	-7.5	-17.8, 2.7	-6.1	-19.9, 7.7	-2.4	-5.2, 0.4
Sarcopenia[e]	0.2	-12.7, 13.0	-3.1	-18.5, 12.3	-5.1	-25.2, 15.0	5.2	-3.8, 14.2	4.4	-9.8, 18.5	-1.9	-21.8, 18.0	3.5	-0.6, 7.5

Abbreviations: CI, confidence interval; HU, Hounsfield units; MD, mean difference; SD, standard deviation; T, tertile.

[a]Adjusted for: age at diagnosis (years), body mass index at health-related quality of life assessment (kg/m^2), number of comorbidities (0/1/2+), cancer stage (I/II/III), and chemotherapy treatment (yes/no).

[b]Scales are 0-100 (global quality of life, physical, role and social functioning, and disability), 20-140 (fatigue), and 0-21 (distress), with higher scores indicating higher global quality of life, physical, role and social functioning, disability, fatigue, and distress. A total of 3 participants had missing data for disability, 2 for fatigue, and 1 for distress. Defined minimal important differences for these subscales are: global quality of life, 10; physical functioning, 14; role functioning, 19; social functioning, 11; disability, 7.7; fatigue, 23.3; and distress, 3.1.

[c]With gender-specific tertiles for visceral adipose tissue (men: T1, ≤100.3; T2, 102.7-196.5; T3, ≥199.2; women: T1, ≤58.7; T2, 59.2-131.0; T3, ≥139.1 cm^2), SMI (men: T1, ≤48.6; T2, 48.8-53.8;

T3, ≥54.0; women: T1, ≤38.7; T2, 38.7-43.9; T3, ≥44.3 cm^2/m^2), and intermuscular adipose tissue (men: T1, ≤8.3; T2, 8.8-14.2; T3, ≥14.3; women: T1, ≤9.6; T2, 9.7-17.8; T3, ≥17.9 cm^2), and overall tertiles for muscle attenuation (T1, ≤33.6; T2, 33.6-41.6; T3, ≥42.0 Hounsfield units).
[d]Dichotomized based on published cutoff for visceral adipose tissue area.[1]
[e]Dichotomized based on published cutoff[2]; data missing for 9 participants due to missing data on body mass index at colorectal cancer diagnosis.

Reference

1. Doyle, S. L., Bennett, A. M., Donohoe, C. L., Mongan, A. M., Howard, J. M., Lithander, F. E., et al. (2013). Establishing computed tomography-defined visceral fat area thresholds for use in obesity-related cancer research. *Nutr Res, 33*(3), 171-179, doi:10.1016/j.nutres.2012.12.007.
2. Martin, L., Birdsell, L., Macdonald, N., Reiman, T., Clandinin, M. T., McCargar, L. J., et al. (2013). Cancer cachexia in the age of obesity: skeletal muscle depletion is a powerful prognostic factor, independent of body mass index. *Journal of Clinical Oncology, 31*(12), 1539-1547, doi:10.1200/JCO.2012.45.2722.
